# The Efficacy and Safety of On-demand Tramadol and Paroxetine Use in Treatment of Life Long Premature Ejaculation: A Randomized Double-blind Placebo-controlled Clinical Trial

**Published:** 2018

**Authors:** Ali Hamidi-Madani, Reza Motiee, Gholamreza Mokhtari, Hamidreza Nasseh, Samaneh Esmaeili, Ehsan Kazemnezhad

**Affiliations:** - Urology Research Center, School of Medicine, Razi hospital, Guilan University of Medical Sciences, Rasht, Iran

**Keywords:** IELT, Paroxetine, PEP, Premature ejaculation, Tramadol

## Abstract

**Background:**

Several medical therapies have been proposed for the treatment of premature ejaculation (PE). Paroxetine and tramadol were both reported to be effective in treatment of PE. In this study, the therapeutic effects of tramadol, paroxetine and placebo were compared in treatment of primary PE.

**Methods:**

In this randomized, double-blind, placebo-controlled clinical trial, 150 patients were divided into 3 groups. One group was treated with tramadol 50 *mg* ondemand, the other group took paroxetine 20 *mg* on-demand and the third group was treated with placebo. Before starting treatment and after 12 weeks, patients were asked to measure their average intravaginal ejaculation latency time (IELT) and fill the PEP (Premature Ejaculation Profile) questionnaire.

**Results:**

At the end of the 12th week, the mean IELT and average of PEP scores improved in all 3 groups. The increase in tramadol group was significantly higher than the paroxetine and placebo groups (p<0.0001). There were no significant differences in terms of side effects between the 3 groups.

**Conclusion:**

The results showed that despite an increase in mean IELT and PEP scores in all 3 groups, the rate of improvement in tramadol group was significantly more than the others. Thus, tramadol may be considered as an appropriate alternative therapeutic option for lifelong PE.

## Introduction

Premature ejaculation (PE) is the most common sexual dysfunction in men (1–2) and may affect many aspects of the life of men, including the loss of confidence, loss of relationships, feelings of anxiety, anger and depression (3). Its prevalence has been reported about 2 to 23% in some studies (4–5). This wide range of prevalence can be caused by different definitions of PE in studies or reflect the differences in diverse populations (6). It seems that all men have experienced PE during their sexual life.

According to the International Society for Sexual Medicine (ISSM), lifelong PE is defined as “a male sexual dysfunction characterized by ejaculation which always or nearly always occurs before or within approximately 1 *min* of vaginal penetration; the inability to delay ejaculation on all or nearly all vaginal penetrations; and negative personal consequences, such as distress, bother, frustration, and/or the avoidance of sexual intimacy” (7).

PE is divided into two categories: primary (lifelong) and secondary or acquired (6, 8). Lifelong PE begins from the onset of sexual maturity and remains as a problem during life. In lifelong PE, ejaculation occurs in less than 1–2 *min* after vaginal penetrating and even before that. Acquired PE develops after an interval of normal sexual function (9). The prevalence of lifelong PE is estimated to be 2–5% and it is 20–30% in acquired PE (10, 11).

Currently, available treatments for PE include selective serotonine re-uptake inhibitors (SSRIs), 1- adrenoreceptor antagonists, the analgesic opioid receptor agonist, antidepressants, local anesthetic agents, phosphodiesterase type 5 inhibitors, *etc*. (6, 8, 12, 13) and each of them has shown varying degrees of effectiveness and side effects (8) and none of them is generally approved for the treatment of PE.

The efficacy of off-label SSRIs in delaying ejaculation with the low side effect makes them the first choice of treatment in PE either on a daily or an on-demand basis (14–16). Paroxetine causes strongest delay in ejaculation compared with other SSRIs. (16) It can be used either on-demand or daily (8, 13, 16). However, the results of the SSRIs’ treatment are not universally successful (17, 18).

Also, the safety and efficacy of tramadol as an analgesic opioid receptor for treatment of lifelong PE has been shown in some recent studies (14, 19–21).

In the present study, the purpose was to compare the safety and efficacy of tramadol, paroxetine and placebo in delaying the intravaginal ejaculation latency time (IELT), improving satisfaction with sexual intercourse and control over ejaculation and decrease ejaculation-related distress and interpersonal difficulty.

## Methods

Between July 2013 and September 2014, a total of 150 healthy men with complaining of lifelong PE who referred to the urology clinic of our university hospital or three other private urology clinics in Rasht enrolled in this double-blind clinical trial.

All men were between 18 to 55 years old, married and had a stable relationship for at least 6 months. PE was defined as uncontrolled ejaculation that occurred before or within 1 *min* of vaginal intercourse. All patients provided a detailed medical and sexual history and physical examination was done for all of them. Exclusion criteria included patients with secondary PE, other sexual dysfunction including erectile dysfunction (ED) according to the international index of erectile function (IIEF), history of a major psychiatric disorder, and endocrine disease (diabetes, liver disease, ...), prostatitis, physical illness or urogenital infection, history of addictive drugs or alcohol abuse, current use of paroxetine or tramadol and sensitivity to this kind of drugs.

An informed consent was obtained from all participants. The study was approved by the local ethics committee of Guilan University of Medical Sciences. It was registered online at Iranian Registry of Clinical Trials (http://www.irct.ir//:IRCT201008304582N2).

IELT and premature ejaculation profile (PEP) questionnaire were used to assess PE. The pretreatment IELT was measured by using a partner-held stop watch during a 3 week baseline period in which patients were asked to experience sexual intercourse at least 3 times. After this initial 3 week screening period, the patients were randomly divided in 3 groups. One group received 50 *mg* tramadol. The other group received 20 *mg* paroxetine and the last group received the placebo. All three pills were identical in appearance and were administered on-demand (2–3 *hr* before the planned intercourse). All patients and investigators were blinded regarding the type of treatment received. Patients were asked to have intercourse at least 6 times and record each IELT throughout these 12 weeks of treatment. Patients were also requested not to use condoms or topical penile anesthetic creams or sprays. At the end of the 12th week, the last 3 IELTs -during the last 3 weeks- for each person were recorded.

The PEP is a validated self-reported questionnaire which contains four items including sexual satisfaction, control over ejaculation, distress and interpersonal difficulty. Each item has 5 possible response options. Each measure regarding ejaculation is scored on a 5 point scale. The PEP questionnaire was filled before and after treatment for all patients.

### Statistical analysis:

According to the normality of data (by Ks test), the IELT between the three groups was compared by Kruskal–Wallis test. The Wilcoxon Rank Test and paired T-test were used for comparing the IELT and PEP scores before and after treatment. The chi-square test was used for comparing the complications between the three groups. A p-value of <0.05 was considered statistically significant.

## Results

A total of 150 patients (range of age, 21–53) were enrolled in this study and randomly divided into 3 groups. Of all the patients, only 126 (84%) cases completed the study period. In the midst of the study, 7(7/4%) patients discontinued due to adverse effects as well as 17 (3/11%) patients (1 case in the paroxetine group and 16 cases in the placebo group) due to the ineffectiveness of treatment. Consequently, the statistical analysis was performed for 126 patients at the end of study.

The mean age of the patients in tramadol group was 36.38±7.92 years; the mean age of the patients in paroxetine group was 35.70±7.89 years, and the mean age of the patients in placebo group was 36.60±6.91 years. The mean age of the patients was similar between the 3 groups (p=0.575).

There was no statistical difference between the groups in terms of baseline IELTs (p=0.368) and PEP scores (p=0.349) (Table 1 and 3).

**Table 1.** Variations of intravaginal ejaculatory latency time (IELT) in different groups

**Characteristics**	**Tramadol**	**Paroxetine**	**Placebo**	p-value^*^
**Baseline IELT**	42.42±17.20	43.20±18.20	47.71±14.37	0.368
**Treatment IELT**	136.98±77.27	91.17±73.22	77.97±54.96	0.0001
**P-value**	0.0001	0.0001	0.001	
**IELT increase (variation)**	94.17±73.97	48.02±68.13	30.47±53.32	0.0001

Significant increase of mean IELT after 12 weeks was observed in all groups (p<0.001). Also, there was statistically significant difference between groups in the improvement of IELT. The increase in the tramadol group (136.98±77.27) was significantly greater than the paroxetine (91.17±73.22) and placebo (77.97±54.96) group (p<0.0001).

A comparison of the increase in the IELTs of three groups after treatment has demonstrated a statistically significant difference (p<0.0001). ELTs changes are shown in table 1.

According to table 2, IELT changes in tramadol group were statistically significant compared to both paroxetine and placebo groups (p<0.0001). But no significant difference was found in paroxetine group versus placebo (p=0.186).

**Table 2.** Change in male intravaginal ejaculation latency time in different groups

	**First group**	**Second group**	**Mean difference**	**SE**	**p-value**	**95% Confidence interval**	
						**Lower Bound**	**Upper Bound**
**Diff-IELT**	Tramadol	paroxetine	46.14	13.85	0.0001	13.29	79.00
		placebo	63.70	15.32	0.0001	27.36	100.04
	paroxetine	placebo	17.55	15.45	0.189	−19.10	54.21

The PEP scores increased from 8.78±2.74 to 13.31± 3.47 in tramadol group, from 9.38±2.55 to 11.30± 3.86 in the paroxetine group and from 9.60±3.01 to 9.97±3.42 in the placebo group (Table 3).

Changes from baseline were significantly greater with tramadol (p<0.0001). All PEP measures improved significantly with tramadol (p<0.0001) and paroxetine groups (p<0.05). Despite the improvement of PEP scores in the placebo group, changes in any of the items were not significant (p>0.05) (Table 4).

**Table 3.** Change in male premature ejaculation profile score in different groups

**Premature Ejaculation Profile Questions**	**Tramadol**	**Paroxetine**	**Placebo**	**p-value**
**Baseline PEP scores % (n)**	8.78±2.74	9.38±2.55	9.60±3.01	0.349
Satisfaction with sexual intercourse^*^				
Control over ejaculation^*^	94 (47)	94 (47)	78 (39)	
Ejaculation-related interpersonal	88 (44)	80 (44)	62 (31)	
Difficulty^**^	22 (11)	18 (9)	26 (13)	
Ejaculation-related distress^**^	52 (26)	66 (33)	50 (25)	
**Treatment PEP scores % (n)**	13.31±3.47	11.30±3.86	9.97±3.42	0.0001
Satisfaction with sexual intercourse^*^				
Control over ejaculation^*^	41.7 (20)	94 (47)	94 (47)	
Ejaculation-related interpersonal	43.8 (21)	88 (44)	88 (44)	
Difficulty^**^	8.4 (4)	22 (11)	22 (11)	
Ejaculation-related distress^**^	23 (11)	52 (26)	52 (26)	
**Diff-PEP**	4.67±4.08	1.98±3.38	0.50±2.64	0.0001

* Very poor or poor;

** Extreme or quite a bit

**Table 4.** Changes in PEP questionnaire’s items before and after treatment in different groups

**Premature Ejaculation Profile Questions**	**Tramadol**	**Paroxetine**	**Placebo**
**Satisfaction with sexual intercourse (Z)**	−5.31	−3.55	−1.10
p-value	0.0001	0.0001	0.270
**Control over ejaculation (Z)**	−5.39	−2.22	−0.09
p-value	0.0001	0.026	0.923
**Ejaculation-related interpersonal difficulty (Z)**	−4.001	−2.34	−1.90
p-value	0.0001	0.019	0.057
**Ejaculation-related distress (Z)**	−3.97	−2.29	−0.81
p-value	0.0001	0.022	0.417

Table 5 shows PEP score changes in tramadol group which was significantly greater than both paroxetine and placebo groups (p<0.0001). But the changes in paroxetine group was not significant compared to placebo (p=0.16).

**Table 5.** Change in male premature ejaculation Profile Score in different groups

	**First group**	**Second group**	**Mean difference**	**SE**	**p-value**	**95% Confidence interval**	
						**Lower bound**	**Upper bound**
**Diff-PEP**	**Tramadol**	paroxetine	2.69	0.72	0.0001	0.97	4.41
		placebo	4.17	0.80	0.0001	2.27	6.07
	**paroxetine**	placebo	1.48	0.81	0.16	−0.44	3.39

No serious adverse effect was observed in the groups. Two patients (4%) in tramadol group reported mild lethargy and drowsiness, 3 patients (6.1%) in paroxetine group had mild fatigue and drowsiness, and 2 patients (5.9%) in placebo group reported mild drowsiness. There was some report about the mild nausea and headache in tramadol group that were tolerated by the patients. Upon comparison of the groups’ reported side effects, there was no significant difference between groups (p=0.879).

## Discussion

Unlike erectile dysfunction (ED), which further affects the older men, PE is the same as occurs in all ages (5, 12). It should be mentioned that PE is not associated with any physiological deficiency and the physiological events of PE are generally normal (22, 23). Because PE has negative effects on quality of sexual life by affecting the satisfaction of the couple, it can be very bothersome. Therefore, the necessity for new treatments for PE is still remaining (23).

The aim of this study was to focus on ejaculation delay and sexual function improvement after ondemand treatment by the measure of IELT and PEP scores together.

In most previous studies, treatment with paroxetine is administered on a daily basis, but in this study, like some others (8, 12, 24) an on-demand (2–3 *hr* before intercourse) oral treatment for lifelong PE was examined.

In the current study, it was shown that the overall efficacy of tramadol is stronger than paroxetine in ejaculation delay during on-demand treatment.

The action of tramadol as a central synthetic opioid analgesic that involves both m-opioid receptor binding and norepinephrine and serotonin reuptake inhibition makes it unique compared to other drugs of the group (14, 23, 25) and because of its acceptable safety can be prescribed for many years (14, 25).

A meta-analysis by Wu et al. (12) showed no difference between tramadol and paroxetine in terms of mean IELT changes (p<0.05). This meta-analysis that has been conducted in various populations concluded that tramadol is a safe and effective treatment for PE.

A successful therapeutic effect of on-demand tramadol in initial increase of the IELTS after 6 weeks compared with the baseline level was reported by Safarinejad and Hosseini (2006) and Salem et al. (2008) (19, 20). A similar finding was reported in 2012 in a study conducted by Kaynar et al. (14). After 8 weeks of treatment, the mean IELT in tramadol group increased significantly compared to the placebo group. In a crossover study by Alghobary et al. (26), the use of daily paroxetine and on-demand tramadol has been evaluated. The mean IELT after 6 weeks significantly increased in both groups, but after 12 weeks, the mean IELT in tramadol group declined while it increased again in paroxetine group compared to sixth weeks. They concluded that the use of daily paroxetine is more effective than tramadol in the treatment of lifelong PE, and tramadol cannot be recommended as a long term treatment.

Bar-Or et al. (21) in a 12-week study compared the therapeutic effects of tramadol with 2 different doses (62 and 89 *mg*) with placebo. The mean IELT increased in all 3 groups, but the increase in the tramadol group (4.2 fold in tramadol 62 *mg* and 2.5 fold in tramadol 89 *mg*) was significantly higher than placebo (6.1 fold) (p<0.001).

In our study, the mean IELT was significantly increased in all groups (p<0.0001). Patients in tramadol group had significantly higher IELT than the other 2 groups (p<0.0001). But changes in mean IELT in paroxetine group was not significant compared to placebo group (p=0.189).

In the present study, efficacy of the treatment was evaluated not only by IELT, but also by measurement of the satisfaction with sexual intercourse, control over ejaculation, personal distress and interpersonal difficulty. Changes in these variables more appropriately indicate the effects of the treatment in men with PE.

Based on Ozcan et al.’s (13) findings, treatment with paroxetine significantly improves the all 4 items of PEP questionnaire compared to the baseline (p<0.001). In another study, significant improvement was observed in all 4 items of PEP questionnaire at 62 and 89 *mg* of tramadol in treatment groups compared to placebo (p<0.05) (21).

In the present study, the mean PEP score increased in all 3 groups. The increase in PEP caused by tramadol was statistically significant compared with the other 2 groups (p<0.0001). The assessment of questionnaire showed that changes in all 4 items have been improved in all 3 groups except the item “concern” in tramadol group which increased significantly. However, these changes were statistically significant only in tramadol and paroxetine group (p<0.0001).

There were no serious adverse effects in groups (p=0.879). The most common side effects were mild lethargy, fatigue, and drowsiness in 3 groups.

Drowsiness is reported in many studies as a major adverse effect for paroxetine and tramadol (14, 24, 27). Also, there were some reports of gastrointestinal distress (26, 27). But the severity level of these side effects was often low.

The low effectiveness of paroxetine compared with other studies may be due to the on-demand usage of this agent in our study, as previous researches have shown that chronic daily treatment with SSRI agents is effective in delaying ejaculation (12, 28).

One of the most important limitations of this study was the large number of patients in placebo group who discontinued the study. A power calculation estimated 50 subjects for each arm to show significance but since our data showed significance with this sample size, the result can be acceptable.

## Conclusion

Our findings indicated that 50 *mg* on-demand tramadol is more effective than on-demand paroxetine 20 *mg* as a long-term treatment of lifelong PE. So, tramadol is expected to be an alternative for the treatment of primary PE. Our study also showed that the use of on-demand paroxetine has no noticeable effect in comparison to placebo. However, further prospective studies with larger sample sizes are needed to conclusively prove these results.
